# LncRNA of peripheral blood mononuclear cells: HYMAI acts as a potential diagnostic and therapeutic biomarker for female major depressive disorder

**DOI:** 10.3389/fpsyt.2025.1241089

**Published:** 2025-02-27

**Authors:** Tianyi Bu, Jiarun Yang, Jiawei Zhou, Yeran Liu, Kexin Qiao, Yan Wang, Jili Zhang, Erying Zhao, Boakye Kwame Owura, Xiaohui Qiu, Zhengxue Qiao, Yanjie Yang

**Affiliations:** ^1^ Psychology and Health Management Center, Harbin Medical University, Harbin, China; ^2^ Department of Psychology, School of Education of Heilongjiang University, Harbin, China; ^3^ School of Humanities, Harbin Medical University, Harbin, China

**Keywords:** major depressive disorder, lncRNA, diagnostic biomarker, high throughput sequencing, bioinformatics analysis, peripheral blood mononuclear cells

## Abstract

**Introduction:**

As a common and complex mental disorder, major depressive disorder (MDD) has brought a huge burden and challenges globally. Although the incidence of female MDD is twice that of male MDD, there are still no accurate diagnostic and treatment criteria for female MDD. The potential of long non-coding RNAs (lncRNAs) as efficient and accurate diagnostic and therapeutic biomarkers provides more possibilities for early and accurate diagnosis of MDD.

**Methods:**

First, the differential expression profile of lncRNAs in peripheral blood mononuclear cells (PBMCs) between MDD patients and healthy controls was established based on high-throughput sequencing analysis. Then, the potential biomarker was screened out by quantifying differentially expressed lncRNAs based on quantitative real-time PCR. To further investigate the function of biomarkers in the pathogenesis of MDD, bioinformatics analysis on downstream target genes was carried out.

**Results:**

The expression profile screened out 300 differentially expressed lncRNAs. HYMAI was proved to be the potential diagnostic biomarker. Its expression levels were significantly higher in MDD patients than in healthy controls with high potential diagnostic value. Based on bioinformatics analysis, a HYMAI–miRNA–mRNA network and a protein–protein interaction network were established, which also showed that HYMAI is closely related to MDD.

**Discussion:**

Our findings showed that the dysregulated expression of lncRNA HYMAI may be the pathophysiological basis of women suffering from MDD. Here, insight into the molecular mechanism of women’s susceptibility to MDD is shown. Meanwhile, a new perspective for future female MDD prevention, diagnosis and treatment, evaluation, detection, and intervention is provided.

## 1 Introduction

Major depressive disorder (MDD) is a common and complex mental disorder with a considerable morbidity rate. It is one of the main causes of disability, disease burden (1), and suicide deaths globally (2, 3). A large-scale longitudinal study has illustrated that MDD increases people’s risk of suffering from other chronic diseases, cancer, and other mental diseases (4). At present, the criteria for the diagnosis and treatment of MDD are mainly based on the signs and symptoms of the diagnosis category. Meanwhile, the diagnostic outcome and treatment plan still depend on the subjective evaluation of the psychiatrists’ clinical experience. Due to the heterogeneity and the unclear evaluation boundary of MDD, MDD is difficult to be accurately diagnosed (5, 6). The assessment of the severity of MDD based on patient symptoms is not accurate enough; as a result, a considerable number of MDD patients fail to receive appropriate treatment, develop resistance to treatment, and have a high recurrence rate (7, 8). Therefore, there is an urgent need to clarify the pathogenesis of MDD and explore efficient, objective, and precise diagnostic and therapeutic biomarkers so as to provide more possibilities for early diagnosis and precise treatment of MDD.

The pathogenesis of MDD needs to be further explored. Previous studies have illustrated that MDD is a multifactorial disorder with multiple neurobiological hypotheses, involving imbalances in levels of monoamine neurotransmitters (dopamine, serotonin, and norepinephrine) in the brain, alterations in neuroplasticity (neurogenesis and synaptic plasticity), dysregulation of the immune system, and the involvement of specific brain regions (the prefrontal cortex, hippocampus, and amygdala) (9, 10). The involvement of epigenetic mechanisms gains increasing attention. Recent advances in gene regulation through epigenetic mechanisms suggest that its regulation of the genome is far beyond what was originally thought (11). Epigenetic factors may not only contribute to the pathogenesis of MDD but may also affect an individual’s response to medication (12). Long non-coding RNA (lncRNA) is a transcript with a length of more than 200 nucleotides and plays a complex regulatory role in gene expression in the form of RNA at the transcriptional, post-transcriptional, and epigenetic levels (13, 14). It has been considered to be the most complex and diverse RNA with functions and types discovered so far, and its quantity is basically the same as the quantity of messenger RNA (mRNA) (14, 15). It is well documented that lncRNAs have taken part in different stages of brain development and its activity, such as being involved in synaptogenesis (16), synaptic plasticity (18), neurogenesis (16), neuron development (17), and maintenance of proper nervous system functioning (18). LncRNA may play a crucial role in the etiology of neurological and psychiatric diseases. Previous studies have shown that epigenetic changes can alter the function and structure of neurons in some way, thereby participating in the occurrence and development of MDD (19). A review of the link between lncRNA and MDD suggests that lncRNA may be the most promising new biomarker for MDD and has the potential to be a therapeutic target (20).

Numerous studies have shown that lncRNA is involved in various key epigenetic regulatory processes and thus participates in the occurrence and development of diseases. According to the competitive endogenous RNA (ceRNA) hypothesis, ceRNA competitively binds microRNA (miRNA) through miRNA response elements (MREs); thereby, the regulation of miRNAs on their target genes will be affected (21). As a ceRNA, the regulatory role of lncRNA has been widely explored in various diseases, such as cancer (22), osteoarthritis (23), diabetes (24), and myasthenia gravis (25). However, very few studies have explored the ceRNA mechanism of lncRNA in MDD. Barry suggested that lncRNAs present as critical regulators in the evolution of higher brain functions (26). Previous studies have also emphasized that 30% of differentially expressed genes in patients with MDD were lncRNAs (27). All lines of evidence showed that the molecular mechanism of the role of dysregulated lncRNAs in MDD needs further investigation.

As we all know, women are twice as likely to suffer from MDD compared with men (28), with higher severity of symptoms, more severe dysfunction, less typical depressive symptoms, and a higher rate of co-morbid anxiety (29). Functionally, the brains of men and women with MDD observe different amplitude, laterality, and volume changes (30). Meanwhile, the responses to the treatment vary with gender (29, 31). However, the molecular mechanisms contributing to this sex difference remain poorly understood till now. Labonté et al. exhibited the transcriptomes of six brain regions of MDD and healthy persons based on RNA-sequencing analysis (32). The result showed that MDD is characterized by strong sexual dimorphism due to hardly any overlap between male and female MDD differential transcripts. Based on the above dataset, Issler et al. carried out a further analysis and pointed out there is a sex-specific regulatory influence of lncRNAs on gene expression (27). Zhou et al. showed that the regulatory influence of lncRNAs on gene expression was stronger in women than men (33). All these findings revealed that male and female MDD may be derived from different genetic changes. In other words, the pathogenesis of MDD is not the same between men and women. It is necessary to explore the pathogenesis of MDD on a gender-specific basis. To summarize, women are a group with a high incidence of MDD, and the pathogenesis not only should be paid more attention but also needs to be explored separately.

The purpose of this study was to explore potential diagnostic biomarkers of female MDD and then investigate its regulatory role in female MDD pathogenesis and development. First, high-throughput sequencing analysis was utilized in three paired female MDD patients and healthy controls (HCs) in order to establish a peripheral blood mononuclear cells (PBMCs) lncRNA expression profile. In addition, five differentially expressed candidate lncRNAs were chosen to evaluate the expression level through quantitative real-time PCR (qRT–PCR) for potential MDD diagnostic biomarker validation (MDD, n = 20; HCs, n = 20) as the preliminary verification step. Subsequently, potential biomarkers with high diagnostic value were selected for further verification in the larger cohorts (MDD, n = 70; HCs, n = 70). Finally, to better explain its diagnostic and therapeutic value, bioinformatics analysis was utilized on validated biomarkers to explore its potential function in the pathogenesis of female MDD.

## 2 Methods

### 2.1 Study subjects

A total of 73 female patients who met the MDD criteria admitted to the First Psychiatric Hospital of Harbin, Harbin, China, between March 2018 and June 2019 were recruited. The diagnosis was independently given by two psychiatrists subject to the diagnostic criteria of the Diagnostic and Statistical Manual of Mental Disorders, Fifth Edition (DSM-5), based on the structured interviews. The 73 HCs who had undergone physical examination during the same period, matched for MDD patients’ age, gender, and ethnicity, were enrolled. In addition, using the 32-item Hypomania Checklist (HCL-32), all the participants were assessed in order to rule out the participants who had previous episodes of mania or hypomania. All the participants who were included in this study met the criteria, as follows: 1) Han ethnicity, 2) age range of 18–60 years, 3) either first-time visitors or had not received any clinical treatment or intervention at least three months, 4) elementary school education or above, 5) without other physical or neurological diseases (such as cardiopathy, diabetes, or Parkinson’s disease), 6) without history of alcohol or drug abuse, 7) without family history of neuropsychiatric disorders, and 8) currently were not in puberty, perimenopause, or pregnancy. The study was approved by the Ethical Committee of Harbin Medical University. All participants provided signed informed consent forms.

### 2.2 Blood sample collection

After overnight fasting, 5 mL of peripheral blood was collected using ethylenediaminetetraacetic acid (EDTA) anticoagulant vacutainers and then centrifuged within 2 hours. The PBMCs were separated from the peripheral blood by density-gradient centrifugation over a human peripheral blood lymphocyte separator according to the manufacturer’s protocol.

### 2.3 RNA extraction in PBMCs

Total RNA was lysed from all the samples with TRIzol reagent (Invitrogen, Carlsbad, CA, USA) according to the manufacturer’s protocol. Three PBMCs samples from the MDD group were randomly collected, and paired three age-, gender-, and ethnicity-matched HCs were prepared for later high-throughput sequencing analysis. All the other samples were frozen at −80°C for later use. NanoDrop ND-2000 spectrophotometer (Thermo Fisher Scientific, Waltham, MA, USA) was used for evaluating RNA concentration. Afterward, all the qualified RNAs were reverse-transcribed into complementary DNA (cDNA) using ReverTra Ace qPCR RT Master Mix (TOYOBO, Osaka, Japan) according to the manufacturer’s instructions and then frozen at −80°C for further qRT–PCR.

### 2.4 High-throughput sequencing analysis

To investigate the expression profile of lncRNAs in MDD, three MDD patients and three matched HCs were analyzed by high-throughput sequencing. Above all, sample concentration and quality testing of PBMCs samples were performed using NanoDrop and Agilent 2100 Bioanalyzer. The sample starting amount needed to reach m ≥ 5 μg, and the sample quality needed to reach c ≥ 200 ng/200 ng/μL and RIN ≥ 7.0 28S/18S ≥ 1.0. Then, the RNase H kit was utilized to remove ribosomal RNA, and the reaction system was configured for RNA fragmentation. Furthermore, the pre-prepared first-strand synthesis reaction mixture was added to the fragmented RNAs in order to synthesize first-strand cDNAs, and then the second-strand synthesis reaction was configured for second-strand cDNA synthesis. After the reaction system and program were configured and set up, double-stranded cDNA fragments were subjected to end-repair, and then a single “A” nucleotide was added to the 3′ ends of the blunt fragments. The reaction system and program for adaptor ligation were subsequently configured and set up to ligate adaptors with the cDNAs. The PCR system was configured to amplify the product. Moreover, the corresponding library quality control protocol was selected depending on product requirements. The fragment range and concentration were detected and quantified using the Agilent 2100 Bioanalyzer and ABI StepOnePlus 156Real-Time PCR System (TaqMan Probe), respectively. Finally, sequencing was performed on the Hiseq 4000 or Hiseq X-ten platform (BGI-Shenzhen, Shenzhen, China). Single-stranded circle DNA molecules were replicated via rolling circle amplification, and a DNA nanoball (DNB), which contains multiple copies of DNA, was generated. Sufficient quality DNBs were then loaded into patterned nanoarrays using a high-intensity DNA nanochip technique and sequenced through combinatorial probe-anchor synthesis (cPAS). The differentially expressed lncRNAs (DElncRNAs) of the HCs group and the MDD group were analyzed using the R studio (V4.0.4) DESeq package. Those that reached *Q*-values < 0.01 and |log_2_FC| > 1 were differential lncRNAs, where those with log_2_FC > 1 were marked as upregulated lncRNAs and those with log_2_FC < −1 were marked as downregulated lncRNAs, and those that do not meet the above conditions were not DElncRNAs.

### 2.5 Quantitative real-time PCR analysis

The candidate DElncRNAs were quantified by qRT–PCR using the Roche LightCycler 480II system (Roche Diagnostics, Basel, Switzerland). The reaction conditions implemented were as follows: denaturation at 95°C for 10 min and 40 cycles of amplification including 95°C for 10 s, 55°C for 10 s, and 72°C for 10 s. All qRT–PCRs were performed in triplicate. Human β-actin was selected for normalizing RNA relative expression level. The expression levels of each lncRNA were calculated by the 2_−ΔΔCt_ method. The ΔCt value equaled the average of each target lncRNA Ct value minus the β-actin Ct value in corresponding samples.

### 2.6 Bioinformatics analysis

#### 2.6.1 Prediction of lncRNA–miRNA–mRNA network

The algorithm DIANA-LncBase v3.0 (https://diana.e-ce.uth.gr/lncbasev3) was applied to predict the target miRNAs of the previously verified lncRNA. The potential targets of these selected microRNAs were predicted using the bioinformatics tool ENCORI (The Encyclopedia of RNA Interactomes; http://starbase.sysu.edu.cn/index.php), miRDB (http://mirdb.org/), miRWalk 3 (Http://mirwalk.umm.uni-heidelberg.de/), TargetScan 7.2 (http://www.targetscan.org/vert_72/), and miRPathDB v2.0 (https://mpd.bioinf.uni-sb.de/overview.html). In order to obtain the intersection of target genes in each database to enhance the reliability of the predicted, Venn diagrams were calculated and drawn using the tool Bioinformatics & Evolutionary Genomics (http://bioinformatics.psb.ugent.be/webtools/Venn/). The constructed lncRNA–miRNA–mRNA network was visualized using the Cytoscape software (V.3.8.2).

#### 2.6.2 GO and KEGG pathway enrichment analysis

In order to exhibit the potential function of the predicted target genes more comprehensively, the Gene Ontology Resource (GO) (http://geneontology.org/) function annotation and Kyoto Encyclopedia of Genes and Genomes (KEGG) (https://www.kegg.jp/kegg/) pathway enrichment analysis of target genes were carried out using DAVID 6.8 (https://david.ncifcrf.gov/). GO function annotation provides an interpretation of target gene function from the following three aspects: molecular functions of gene products, the cellular environment they are located in, and the biological processes. The terms were screened out with *p* < 0.05, which showed significantly enriched functions.

#### 2.6.6 Construction PPI network and module analysis

To further illuminate the function of predicted genes in the ceRNA network, a protein–protein interaction (PPI) network was established using Search Tool for the Retrieval of Interacting Genes (STRING Version 11.0; https://string-db.org/). In the Cystoscope software, the Molecular Complex Detection (MCODE) plug-in was used for gene cluster classification, and the CytoHubba plug-in was used for hub gene filtering. At last, the PPI network was constructed and visualized using the Cytoscape software (V.3.8.2).

### 2.7 Statistical analysis

Volcano plot and heat map were visualized using differentially expressed genes with the ggplot2 package in R version 4.0.3. All statistical analyses were conducted using the GraphPad 8 software (Version 8.0.2; GraphPad Software, La Jolla, CA, USA) and R software (Version 4.0.3). Data distributions were assessed by the Shapiro–Wilk test. If the *p*-value was greater than 0.05, the data were considered to be in a normal distribution; otherwise, the data were considered to be non-normal. The differences in the expression levels of lncRNAs among patients with MDD and HCs were assessed using a t-test if the data were normally distributed. Otherwise, the Mann–Whitney U test was used. Findings with *p* < 0.05 results were considered statistically significant. To evaluate the diagnostic value of the DElncRNAs, a receiver–operator characteristic (ROC) curve was established. The evaluation of diagnosis ability depended on the area under the ROC curve (AUC). In the case of AUC > 0.5, the closer the AUC value was to 1, the better the diagnostic effect.

## 3 Results

### 3.1 Identification of lncRNA expression profile

**Figure 1:**
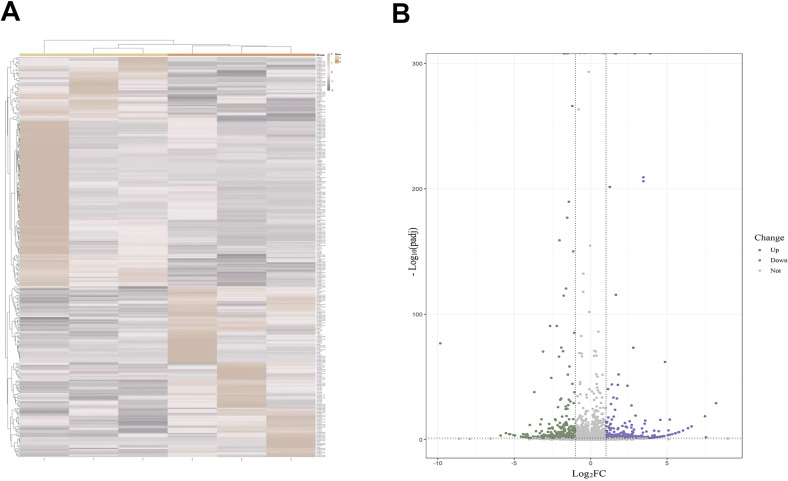
**(A)** Heat map of DElncRNAs associated with MDD. **(B)** Volcano plot highlighting significantly upregulated (purple) and downregulated (green) lncRNAs. DElncRNAs, differentially expressed lncRNAs; lncRNAs, long non-coding RNAs; MDD, major depressive disorder.

Based on high-throughput sequencing analysis, the full expression profile of lncRNAs in PBMCs between two groups was established. On account of threshold |log_2_FC| > 1 and *Q*-value < 0.01 criteria, there were 300 DElncRNAs screened, of which 128 were upregulated and 172 were downregulated as shown in the heat map and volcano plot (**Figures 1A, B**). Afterward, five candidate lncRNAs were chosen for further validation in order to investigate the potential diagnostic biomarker. The primers for qRT–PCR are listed in **Table 1**.

**Table 1:** Primers used for qRT–PCR analysis of lncRNA expression levels.

Target ID	Primer sequence (5′–3′)
HYMAI	F: TTGAAAGCCACTGCCCTAGA
HYMAI	R: CTTCAGGTAGGCATGAAGGC
LOC107987438	F: TGCGGTAATGGACCAAGAGT
LOC107987438	R: CTCTGAGAAGGGCGAGTGAT
LOC105377781	F: GTCCTGAAAGACCAGGTCCA
LOC105377781	R: ATGCAGTTCCTGCCTCTCAT
LOC105373656	F: AGGCTCAACTCCGAATGGAT
LOC105373656	R: GGTCCAAGCCAAGCCAAAC
LOC107987461	F: TCAACAAAGCCAGTGAAGCTC
LOC107987461	R: ACGATCTGTAGGGCTGTCTG
β-Actin (human)	F: GGGAAATCGTGCGTGACATT
β-Actin (human)	R: GGAACCGCTCATTGCCAAT

lncRNA, long non-coding RNA.

### 3.2 Validation of the diagnostic biomarker

**Figure 2:**
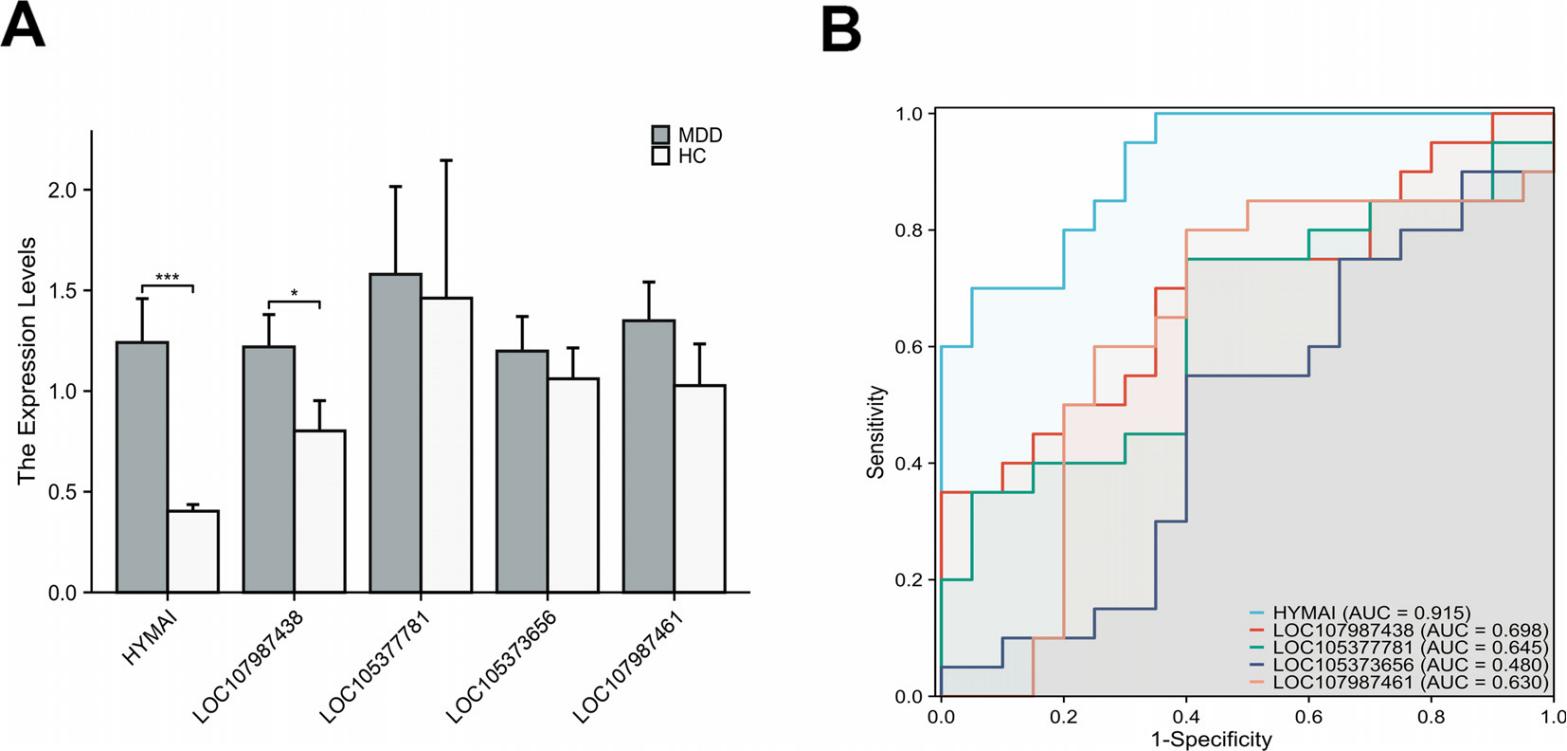
The differential expression levels and ROC curves of DElncRNAs in PBMCs between MDD patients (MDD, n = 20) and healthy controls (HCs, n = 20) by qRT–PCR. **(A)** The expression of HYMAI and LOC107987438 in MDD was significantly higher than that in HCs. *p < 0.05,***p < 0.001. **(B)** ROC curve analysis of HYMAI, LOC107987438, LOC105377781, LOC105373656 and LOC107987461. ROC, receiver operating characteristic; DElncRNAs, differentially expressed long non-coding RNAs; PBMCs, peripheral blood mononuclear cells; MDD, major depressive disorder.

**Figure 3:**
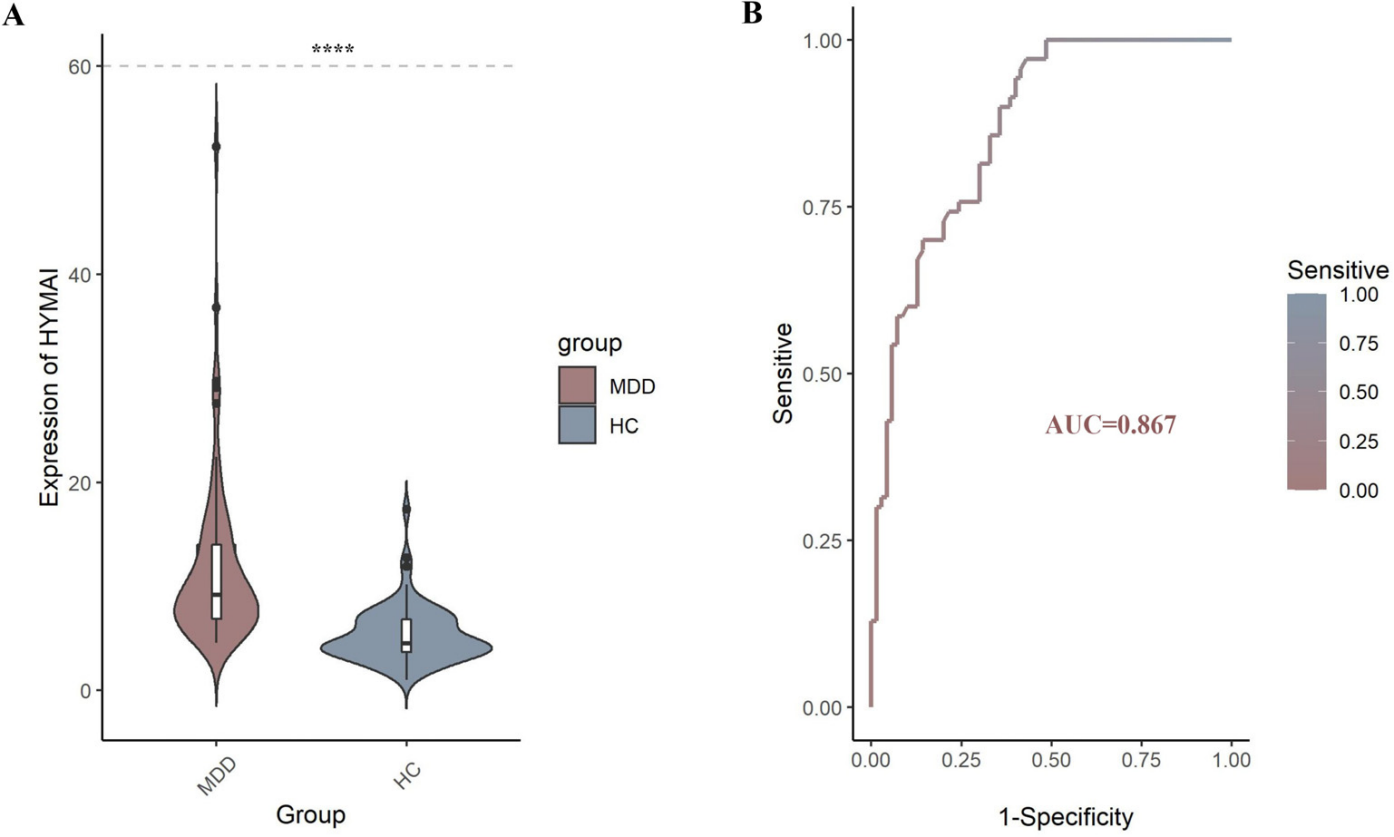
**(A)** The differential expression levels of lncRNA HYMAI in PBMCs between two groups (MDD, n = 70; HCs, n = 70). HYMAI expression level was increased in MDD group compared with HCs group, as determined by qRT–PCR. ****p < 0.0001. **(B)** The ROC curves of lncRNA HYMAI in PBMCs of MDD group. The area under the ROC curve was 0.867. lncRNA, long non-coding RNA; PBMCs, peripheral blood mononuclear cells; MDD, major depressive disorder; ROC, receiver operating characteristic.

The expression levels of DElncRNAs in PBMCs between female MDD patients and HCs were quantified by qRT–PCR. In the preliminary validation step (MDD n = 20, HCs n = 20), the levels of HYMAI and LOC107987438 expression were significantly higher in patients with MDD than in healthy controls (**Figure 2A**). The diagnostic values of these two candidate biomarkers were evaluated using ROC curves. Based on ROC curve analysis, HYMAI presented a better diagnostic value because of the larger AUC (AUC = 0.915, 95% CI: 0.834–0.996, *p* < 0.05) compared to LOC107987438 (AUC = 0.698, 95% CI: 0.531–0.864, *p* = 0.097) (**Figure 2B**). To further determine the actual diagnostic value of HYMAI, a larger cohort qRT–PCR was carried out. In the further validation step, the expression of lncRNA HYMAI remained significantly different. The expression levels of lncRNA HYMAI were significantly higher in patients with MDD than in HCs (**Figure 3A**). The AUC of lncRNA HYMAI was 0.867 (95% CI: 0.809–0.925, *p* < 0.0001), suggesting it has a high potential diagnostic value (**Figure 3B**).

### 3.3 Interaction of lncRNA–miRNA–mRNA

**Figure 4:**
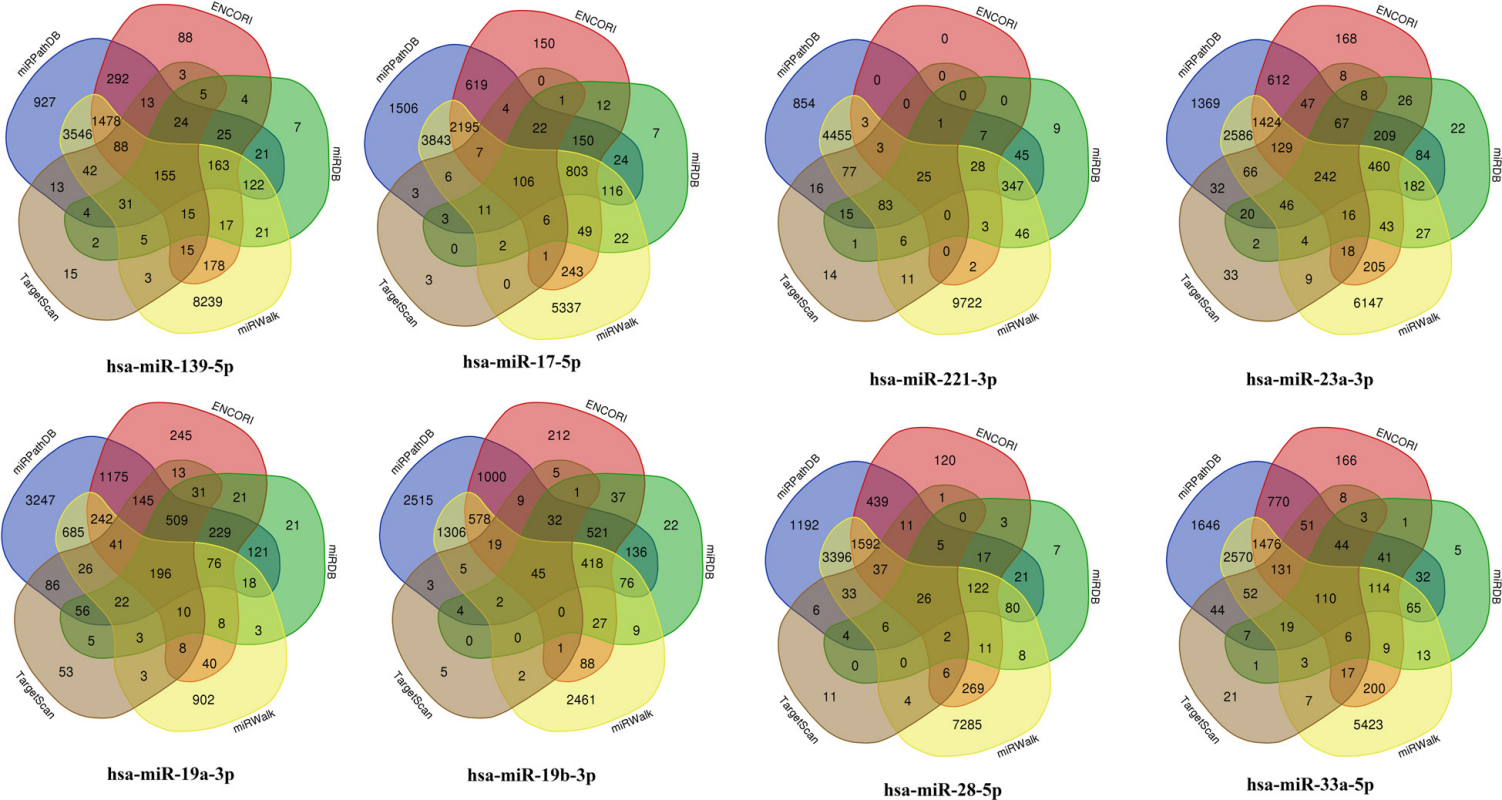
Venn diagram of the predicted miRNA target genes. miRNA, microRNA.

**Figure 5:**
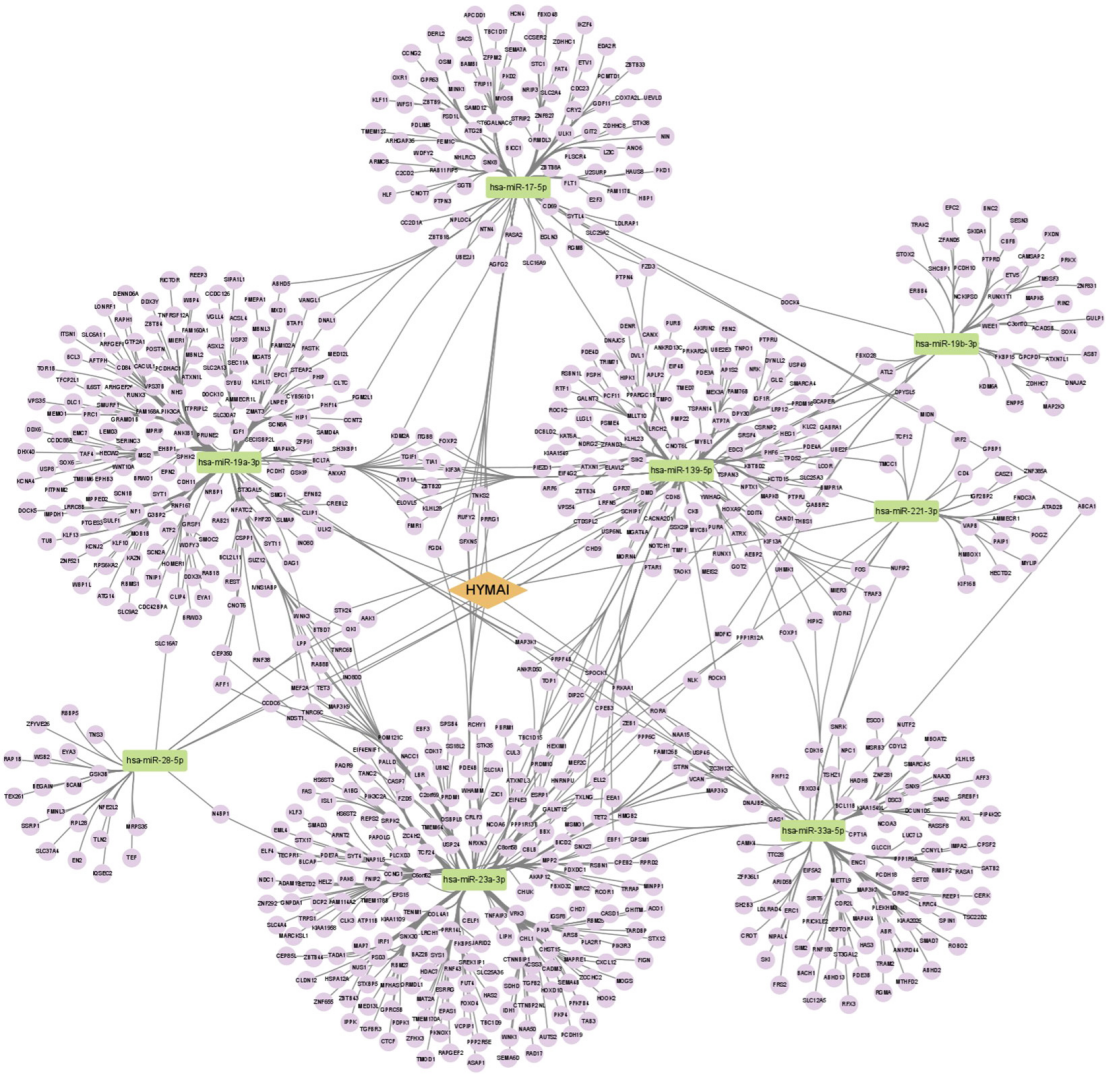
ceRNA network of lncRNA HYMAI–miRNA–mRNA in female major depressive disorder. The network consists of 1 lncRNA node, 8 miRNA nodes, and 897 mRNA nodes. Orange color indicates lncRNA, green color presents miRNA, and purple color illustrates mRNA. lncRNA, long non-coding RNA; miRNA, microRNA; ceRNA, competitive endogenous RNA; mRNA, messenger RNA.

A total of nine miRNAs were predicted to target HYMAI by LncBase: hsa-miR-139-5p, hsa-miR-17-5p, hsa-miR-19a-3p, hsa-miR-19b-3p, hsa-miR-221-3p, hsa-miR-23a-3p, hsa-miR-28-5p, hsa-miR-33a-5p, and hsa-miR-23b-3p. Among these nine miRNAs, only hsa-miR-23b-3p presents low miRNA confidence. To further examine the potential downstream pathway, the remaining eight high-confidence miRNAs were selected to predict target genes. The target genes after taking the intersection were displayed in the Venn diagram (**Figure 4**). A total of 897 target genes harbored the above eight miRNAs. Subsequently, the constructed lncRNA HYMAI–miRNA–mRNA network map is presented in **Figure 5**. hsa-miR-23a-3p exhibited the largest interaction network.

### 3.4 GO and KEGG pathway enrichment analysis

**Figure 6:**
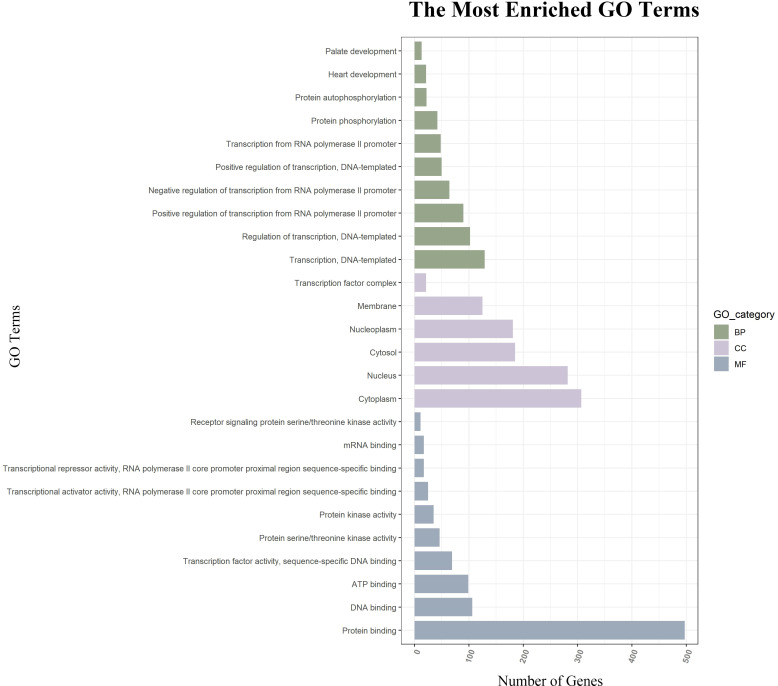
GO function annotations of downstream target genes. The green bar chart illustrates the biological process, the purple car chart expounds the cellular component, and the gray bar chart illustrates the molecular function. GO, Gene Ontology.

**Figure 7:**
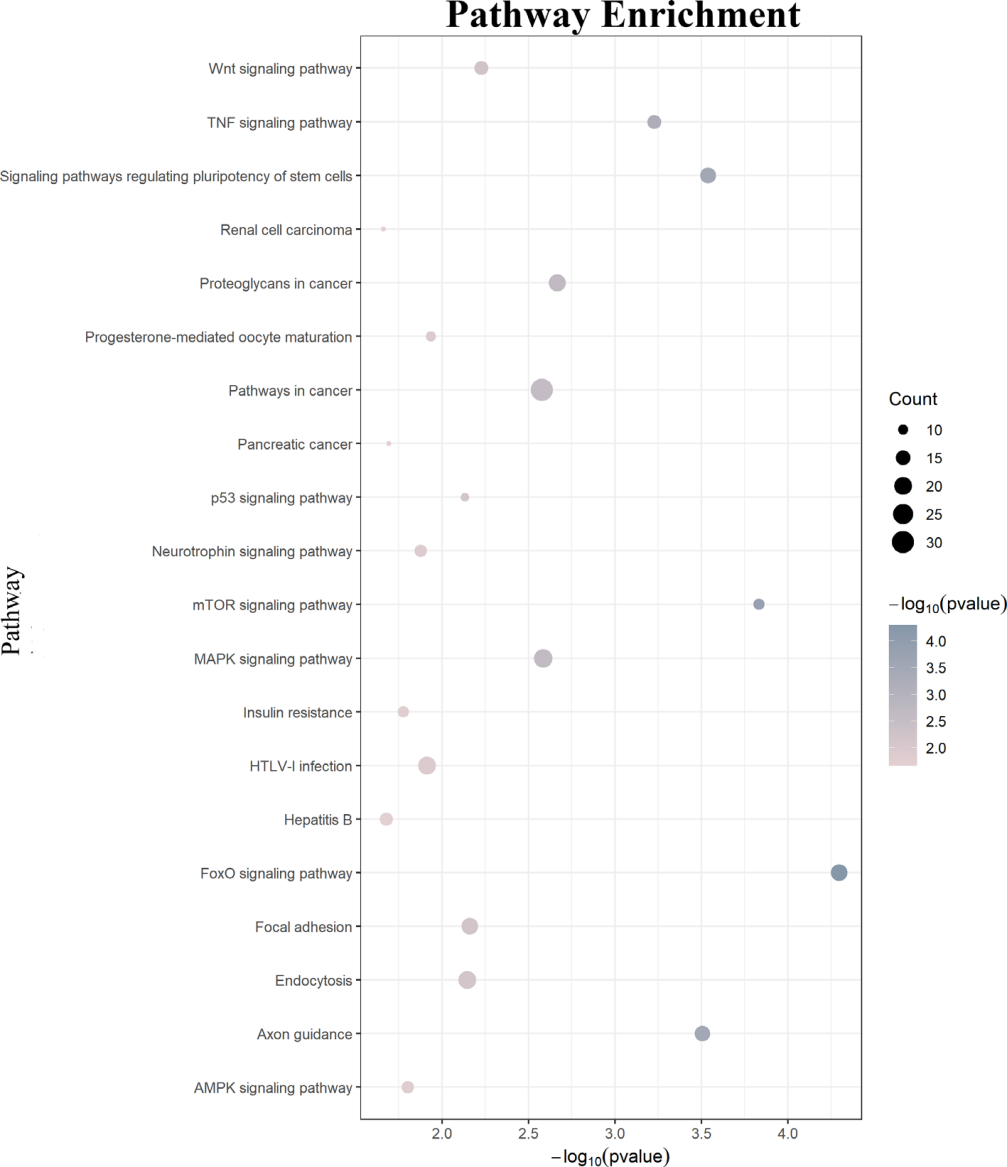
KEGG pathway analysis of the downstream target genes. KEGG, Kyoto Encyclopedia of Genes and Genomes.

GO function annotations and KEGG pathway analysis were carried out for the target genes. Out of the GO function annotations indicated among the 214 biological process terms, the highest enriched terms were “transcription, DNA-templated“, “regulation of transcription, DNA-templated“, and “positive regulation of transcription from RNA polymerase II promoter“ (*p* < 0.05). In terms of cellular components, the target mRNAs were most enriched in terms of “cytoplasm”, “nucleus“, and “cytosol“ (*p* < 0.05). For molecular function, the enriched terms were “protein binding“, “metal ion binding“, and “metal ion binding“ (*p* < 0.05) (**Figure 6**). According to the result of the KEGG pathway analysis, 34 pathways were significantly enriched, such as “FoxO signaling pathway“, “mTOR signaling pathway“, “MAPK signaling pathway“, “Wnt signaling pathway“, “Neurotrophin signaling pathway”, and “Insulin resistance”. The top 20 enriched pathways are shown in **Figure 7**.

### 3.5 Construction PPI network and module analysis

**Figure 8:**
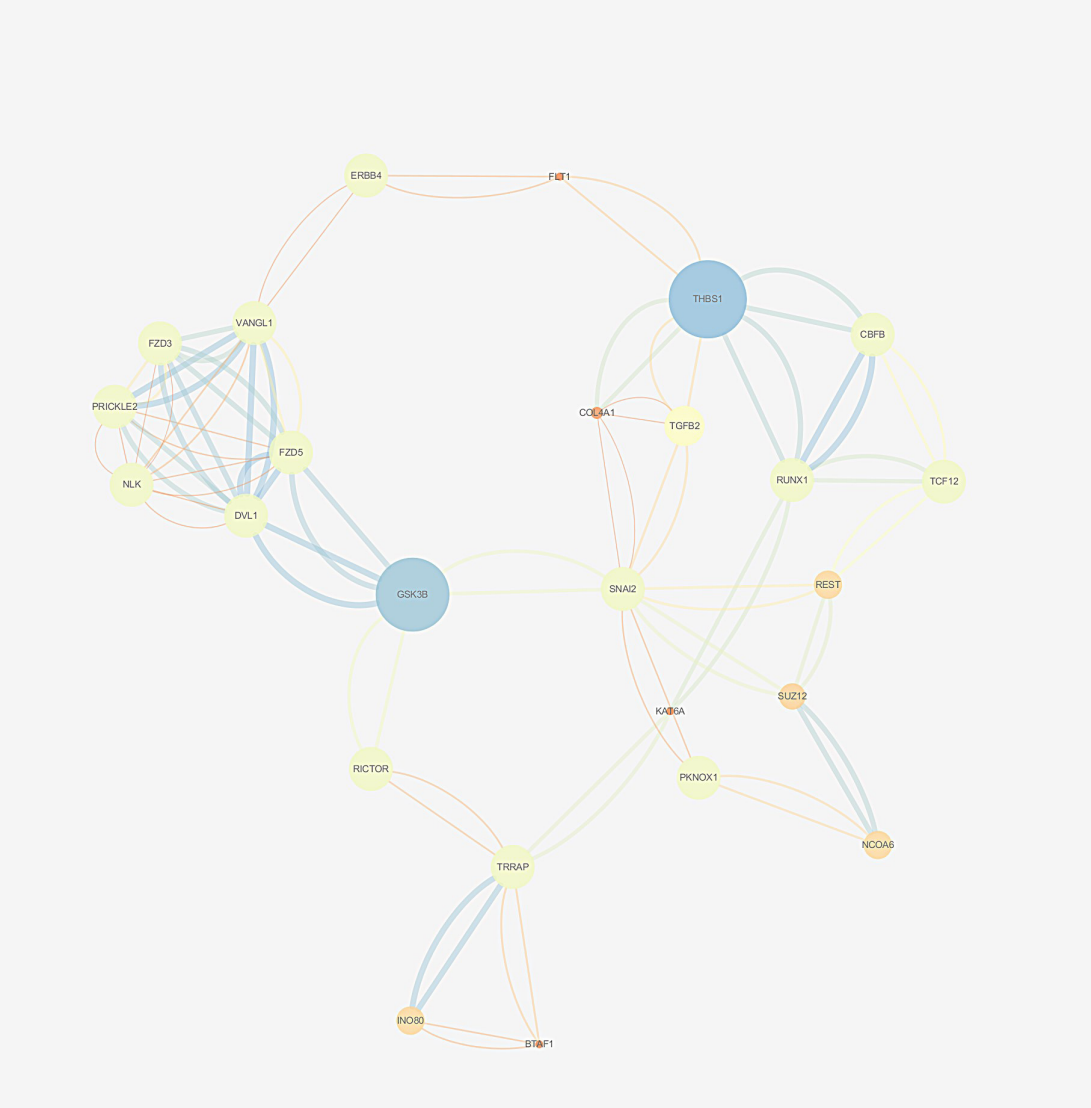
The selected cluster of protein–protein interaction network identified by MCODE was visualized using Cytoscape v.3.6.1. The size of node depends on significance of *p*-value. The more significant *p*-value, the larger the diameter of node. The color of the edge represents the value of combined score from 0.4 to 1, light to dark.

Based on the String database, a PPI network with 800 nodes and 2977 edges was established, which is too huge to show the specific information. The divided module was identified by MCODE from the PPI network. The significant module related to MDD contained 27 nodes and 89 edges, as shown in **Figure 8**. Next, the top 10 hub genes were screened out using CytoHubba based on the Maximal Clique Centrality (McC) method. They were SNAI2, GSK-3β, DVL1, FZD5, THBS1, VANGL1, RUNX1, COL4A1, TGFβ2, and REST.

To sum up, we found that there were a large number of DElncRNAs between the PBMCs of female MDD patients and HCs. HYMAI was validated to have the potential to be a diagnostic and therapeutic biomarker. Based on bioinformatics analysis, a HYMAI–miRNA–mRNA network was established, which also showed that HYMAI is closely related to MDD. Moreover, protein–protein interaction analysis screened out 10 hub genes, which provided the possibility for us to further interpret the mechanism of HYMAI in female MDD.

## 4 Discussion

This study attempted to present a more comprehensive understanding of female MDD pathogenesis. Epigenetic mechanisms as the basis for gene regulation are ideal for studying MDD. At present, little is known about the potential role of lncRNA as a diagnostic and therapeutic biomarker, and there are few studies evaluating the clinical value of lncRNA in efficacy as well. In order to better explore the mechanism of action of lncRNA regulation in MDD, a female-specific lncRNA expression profile in PBMCs between MDD and HCs was established based on high-throughput sequencing analysis. Most previous studies have only explored the differential expression of lncRNA in female MDD patients and HCs based on microarray analysis. In this study, the expression profile of lncRNA was constructed based on high-throughput sequencing, which not only found more novel lncRNA but also laid a foundation for the molecular mechanism study of female MDD. Undoubtedly, for the diagnosis and treatment of MDD, the screening of potential diagnostic markers of MDD by DElncRNA expression profiling based on PBMCs has shown good clinical application value.

As well known, there is no objective physiological and biochemical indicator for MDD, which brings great trouble to the accurate diagnosis and treatment of MDD. Our study explores this question and verifies that lncRNA HYMAI in PBMCs could be the potential diagnostic biomarker for female MDD. MDD peripheral blood biomarkers have incomparable advantages in the application of clinical diagnosis. The discovery of female MDD biomarker lncRNA HYMAI in PBMCs may have some application prospects in the future to help researchers accurately diagnose, monitor treatment response, detect intervention effects, or detect early recurrence in real time. In our previous study, we found that circRNA hsa_circ_0126218 has the potential to become a biomarker of female MDD (34). Compared with previous findings, a higher potential diagnostic value has been shown by lncRNA HYMAI, which suggested that lncRNA HYMAI is more likely to become a biomarker of female MDD. It has been investigated in several fields. Arima et al. suggested that HYMAI as an imprinted gene may participate in transient neonatal diabetes mellitus (35, 36). Another study pointed out that imprinted expression of HYMAI in skin fibroblasts is tight in healthy individuals but not in transient neonatal diabetes mellitus patients (37). Even though HYMAI has been found over-expressed in transient neonatal diabetes mellitus patients, the function of this transcript is still unclear. Thereby, another study was carried out for a deeper interpretation of its molecular mechanism. The study showed that HYMAI may interact with chromatin machinery to protect paternal alleles from DNA methylation (38). More than that, there is also a close link between altered methylation levels at PLAGL1/HYMAI differentially methylated regions and high-risk human papillomavirus infections (39). Ryan et al. indicated that HYMAI expression in the whole blood of patients who acquired chronic inflammatory response syndrome following an exposure to the marine toxin ciguatoxin was significantly upregulated (40). The upregulated HYMAI may also be considered the potential biomarker for endothelial hypoxia in human atherosclerotic lesions (41). A recent study expounded that HYMAI as an autophagy-related lncRNA may play a complex function and could be considered a favorable prognostic factor in acute myeloid leukemia progression (42). In clear cell renal cell carcinoma patients, HYMAI expression has also been found to be associated with overall survival (43). Previous studies have shown that MDD is common among kidney cancer patients (44, 45), atherosclerotic lesion patients (46), diabetes patients (47), acute myeloid leukemia patients (48, 49), and human papillomavirus infection patients (50). There is also a certain correlation between diabetes and coronary artery disease atherosclerotic plaque characteristics (51). Thus, it can be concluded that HYMAI may share genetic vulnerability contributing to the link between MDD and other diseases. Meanwhile, it is also indicated that lncRNA HYMAI could be a potential intervention target for MDD in the future.

As mentioned above, due to the unclear pathogenesis of MDD, there is no effective antidepressant medication, resulting in a high rate of medicine resistance or recurrence. To further interpret the molecular mechanisms of elevated HYMAI in the pathologies of female MDD, bioinformatics analysis was utilized. The established HYMAI–miRNA–mRNA, which provides an intuitive way to analyze the downstream pathway, could promote further analysis of the role of HYMAI in the pathogenesis of MDD. As mentioned above, a total of eight miRNAs with high confidence have been taken into account in the ceRNA network. Four of them (hsa-miR-221-3p, hsa-miR-139-5p, hsa-miR-17-5p, and hsa-miR-19a-3p) have been reported to be closely linked with MDD. A previous study found that the relative expression levels of miR-221-3p, miR-33a-5p, and miR-139-5p in the cerebrospinal fluid of MDD patients were significantly higher than those in HCs based on the microRNA PCR panel analysis. Among them, miR-221-3p may be a potential biomarker for MDD due to its dysregulation in both the cerebrospinal fluid and serum of MDD patients (52). Meanwhile, the expression of serum miR-221-3p is sensitive to depressed mood in perioperative patients as well (53). Furthermore, upregulated exosome-derived miR-139-5p could cause depressive-like behaviors in mice due to its role in the impairment of adult hippocampal neurogenesis (54). Meanwhile, exosomal miR-139-5p was also considered to be the MDD biomarker on account of its dysregulation in MDD patients (55). The expression of miR-19a-3p in PBMCs was reported to be upregulated in MDD patients with suicidal ideation (46). Carmkurt et al. pointed out that the expression level of hsa-miR-17-5p is upregulated in the plasma of patients with MDD (56). Based on a systematic review and a bioinformatics analysis, Ferrúa et al. illustrated that hsa-miR-17-5p is one of the most frequent miRNAs that take part in MDD-related pathways (57). Despite that the other four RNAs have not been reported, they are also worthy of further verification in subsequent studies. Due to the large number of predicted downstream target genes, bioinformatics analysis will be used to further clarify the regulatory network that is more likely to play a role in the pathogenesis of MDD.

The result of target gene function annotation and enriched analysis illustrated that the altered HYMAI is engaged in affecting some important genes and pathways related to MDD, such as “mTOR signaling pathway“, “MAPK signaling pathway“, “Wnt signaling pathway“, and “Insulin resistance’. The mTOR signaling pathway takes part in synaptic protein synthesis (58). Synaptic deficits caused by abnormal dysregulation of mTOR signaling may play a pivotal role in the pathogenesis of MDD (59, 60). The role of the MAPK pathway in MDD has been widely investigated (61, 62). Accumulating biological evidence indicates that depressive-like behaviors contribute to inhibiting the MAPK pathway (8, 63). It is worth noting that a gender-specific study exploring the role of the Ras–Raf–MAPK signaling pathway in MDD revealed that different haplotypes and gene–gene interactions in the pathway may lead to female patients being more susceptible to antidepressant efficacy compared to male patients (64). The dysregulation of the Wnt signaling pathway can lead to various neurological and neuropsychiatric disorders (65, 66). It is widely known that the Wnt signaling pathway participates in MDD (67, 68), as well as in response to antidepressants (69). The relationship between MDD and insulin resistance has gained more and more attention (70, 71). The synergy of insulin resistance and high levels of stress can increase the susceptibility to MDD (72). The results suggest that HYMAI can participate in MDD pathogenesis by regulating downstream target genes.

The established PPI network presented the interactions between the predicted target genes intuitively. The top 10 hub genes are SNAI2, GSK-3β, DVL1, FZD5, THBS1, VANGL1, RUNX1, COL4A1, TGFβ2, and REST. Some of the genes have been suggested to influence the pathophysiology of MDD (Duda et al., 2020). A recent review showed that the age of onset and severity of MDD are closely related to abnormal GS-3β activity, changes in its expression profile, and genetic polymorphisms. It is not only proposed that GSK-3β is an important factor involved in the pathogenesis of MDD but also pointed out the possibility of GSK-3β as a therapeutic target. A recent study found that the dysregulated miR-128-3p in the amygdala of rats could trigger depressive-like behavior by regulating the downstream target genes (Wnt3, Wnt3a, Wnt5b, Dvl1, and Lef1) involved in the Wnt signaling pathway (73). In addition, DVL1 could encode a cytoplasmic scaffolding protein, which has been implicated in THBS1 antidepressant response in rodents (74). RUNX1 has been clarified to act at multiple levels during the nervous system development (75). Wuchty et al. indicated that RUNX1 as a driver causal gene in MDD may play a crucial role in stress- and trauma-related pathways (76). It has been suggested that REST may be involved in the context of mood disorders and the action of antidepressants based on the analysis of its target genes (69). They also mentioned that the expression level of REST is reduced in MDD patients. These findings will facilitate the investigation of mechanisms of women’s susceptibility to MDD. In our study, the highlighted hub genes in the network will elucidate the mechanism of action of lncRNA HYMAI in the occurrence and development of MDD. In turn, a more accurate strategy for the diagnosis and treatment evaluation of MDD could be provided in the future.

Even though our findings will increase the interpretation of the function of lncRNAs in MDD, some limitations still need to be noticed. First of all, the regulation mechanism of HYMAI has been explained based on the ceRNA hypothesis in this study. However, lncRNAs can also be involved in post-transcriptional regulation through a variety of other mechanisms, including interactions with RNA binding proteins, base pairing with mRNA, and interactions with specific organelles. These mechanisms affect mRNA splicing, stability, translation, and signaling pathways, which regulate gene expression. Other functions of HYMAI based on the role of LncRNA, such as regulating gene expression through interaction with key transcription factors, need to be further explored in MDD. Second, this study only explored female MDD. In subsequent studies, we can construct expression profiles for men and explore the differences in the pathogenesis of MDD between different genders. The reasons why women are more likely to suffer from MDD than men may be elaborated. Moreover, the sample size of this study was 70 pairs. To more powerfully determine the biomarkers, a larger and wider population should be considered in future studies. Furthermore, the samples in this study are mainly from human PBMCs, and the functional research of HYMAI should be further strengthened in cell and animal models in the future. Finally, although this study predicted the pathogenesis of female MDD and found a potential therapeutic target, there is still a need for further experimental studies for verification.

## 5 Conclusion

In summary, a female-specific lncRNA expression profile in PBMCs between MDD and HCs was established based on high-throughput sequencing analysis, which would provide a basis for sex-specific exploration of the role of lncRNAs in MDD. Through experimental verification, we found that lncRNA HYMAI in PBMCs has the potential to be used as a biomarker of female MDD and has a good clinical application prospect. According to bioinformatics analysis, it is finally clarified that the dysregulated expression of lncRNA HYMAI may be the pathophysiological basis of women suffering from MDD, which provides a more theoretical basis for exploring the pathogenesis of female MDD. Our findings contribute to providing a new perspective for future MDD prevention, diagnosis and treatment, evaluation, detection, and intervention. In the future, the specific molecular mechanism of lncRNA HYMAI in female MDD should be further explored in order to achieve gender-based precise diagnosis and treatment of MDD.

## Data Availability

The datasets presented in this study can be found in online repositories. The names of the repository/repositories and accession number(s) can be found below: https://www.ncbi.nlm.nih.gov/, PRJNA698421.
